# miR-20b and miR-451a Are Involved in Gastric Carcinogenesis through the PI3K/AKT/mTOR Signaling Pathway: Data from Gastric Cancer Patients, Cell Lines and Ins-Gas Mouse Model

**DOI:** 10.3390/ijms21030877

**Published:** 2020-01-29

**Authors:** Greta Streleckiene, Ruta Inciuraite, Simonas Juzenas, Violeta Salteniene, Ruta Steponaitiene, Ugne Gyvyte, Gediminas Kiudelis, Marcis Leja, Paulius Ruzgys, Saulius Satkauskas, Eugenija Kupcinskiene, Sabine Franke, Cosima Thon, Alexander Link, Juozas Kupcinskas, Jurgita Skieceviciene

**Affiliations:** 1 Institute for Digestive Research, Academy of Medicine, Lithuanian University of Health Sciences, Kaunas LT-50161, Lithuania; greta.streleckiene@lsmuni.lt (G.S.); ruta.inciuraite@lsmuni.lt (R.I.); simonas.juzenas@lsmuni.lt (S.J.); violeta.salteniene@lsmuni.lt (V.S.); ruta.steponaitiene@lsmuni.lt (R.S.); ugne.gyvyte@lsmuni.lt (U.G.); gediminaskiudelis@gmail.com (G.K.); j_kupcinskas@yahoo.com (J.K.); 2 Institute of Clinical Molecular Biology, Christian-Albrechts-University of Kiel, 24105 Kiel, Germany; 3 Institute for Clinical and Preventive Medicine, University of Latvia, Riga LV-1586, Latvia; marcis.leja@lu.lv; 4 Faculty of Medicine, University of Latvia, Riga LV-1586, Latvia; 5 Department of Research, Riga East University Hospital, Riga LV-1038, Latvia; 6 Digestive Diseases Centre GASTRO, Riga LV-1079, Latvia; 7 Biophysical Research Group, Faculty of Natural Sciences, Vytautas Magnus University, Kaunas LT-44404, Lithuania; paulius.ruzgys@vdu.lt (P.R.); saulius.satkauskas@vdu.lt (S.S.); 8 Department of Biology, Faculty of Nature Sciences, Vytautas Magnus University, Kaunas LT-44404, Lithuania; eugenija.kupcinskiene@vdu.lt; 9 Institute of Pathology, Otto-von-Guericke University, 39120 Magdeburg, Germany; sabine.franke@med.ovgu.de; 10 Department of Gastroenterology, Hepatology and Infectious Diseases, Otto-von-Guericke University, 39120 Magdeburg, Germany; cosima.thon@med.ovgu.de (C.T.); alexander.link@med.ovgu.de (A.L.); 11 Department of Gastroenterology, Academy of Medicine, Lithuanian University of Health Sciences, Kaunas LT-50161, Lithuania

**Keywords:** microRNAs, miR-20b, miR-451a, PI3K/AKT/mTOR signaling pathway, gastric cancer

## Abstract

Gastric cancer (GC) is one of the most common and lethal gastrointestinal malignancies worldwide. Many studies have shown that development of GC and other malignancies is mainly driven by alterations of cellular signaling pathways. MicroRNAs (miRNAs) are small noncoding molecules that function as tumor-suppressors or oncogenes, playing an essential role in a variety of fundamental biological processes. In order to understand the functional relevance of miRNA dysregulation, studies analyzing their target genes are of major importance. Here, we chose to analyze two miRNAs, miR-20b and miR-451a, shown to be deregulated in many different malignancies, including GC. Deregulated expression of miR-20b and miR-451a was determined in GC cell lines and the INS-GAS mouse model. Using Western Blot and luciferase reporter assay we determined that miR-20b directly regulates expression of *PTEN* and *TXNIP,* and miR-451a: *CAV1* and *TSC1*. Loss-of-function experiments revealed that down-regulation of miR-20b and up-regulation of miR-451a expression exhibits an anti-tumor effect in vitro (miR-20b: reduced viability, colony formation, increased apoptosis rate, and miR-451a: reduced colony forming ability). To summarize, the present study identified that expression of miR-20b and miR-451a are deregulated in vitro and in vivo and have a tumor suppressive role in GC through regulation of the PI3K/AKT/mTOR signaling pathway.

## 1. Introduction

Gastric cancer (GC) is one of the most prevalent malignancies and a leading cause of cancer-related mortality worldwide [1]. The development and progression of GC is a multistep process involving accumulation of genetic mutations and alterations in proto-oncogenes or tumor-suppressor genes [2]. One of the major issues in clinical settings of GC is a poor survival rate and prognosis of patients, as the tumor is diagnosed at late stages of the disease. Therefore, further insight into the molecular mechanisms underlying GC progression may help to identify novel therapeutic targets and improve the prognosis of GC.

MicroRNAs (miRNAs) are small noncoding molecules that, being part of a so-called RNA-induced silencing complex (RISC), post-transcriptionally regulate gene expression [3]. Dependent on the target gene and background conditions, miRNAs may function as either tumor-suppressors which suppress protein-coding oncogenes, or as oncogenic miRNAs which negatively regulate known tumor-suppressor genes [4]. In this way they play an essential role in a variety of fundamental biological and pathological processes [3]. Moreover, miRNA-based therapy is being tested as a potential strategy in cancer treatment [5,6,7,8]. A number of GC studies have reported specific signatures of deregulated miRNAs belonging to miR-17, miR-19, miR-21, miR-223, miR-135, and other families, and their diagnostic and prognostic potential [9,10,11,12]. However, in order to understand functional relevance of miRNA dysregulation, studies analyzing their target genes are of major importance.

Here, we chose to analyze two miRNAs, miR-20b and miR-451a, shown to be deregulated in many different malignancies, including GC [13,14,15,16,17]. Previous functional studies have revealed that these miRNAs may exert their biological role through mediating tumor formation, maintenance, and metastasis [18,19,20,21]. However, the role of these miRNAs and possible target genes in GC remain poorly investigated. In this study, we found that inhibition of miR-20b and overexpression of miR-451a had a tumor-suppressive role in GC through the regulation of genes involved in the PI3K/AKT/mTOR signaling pathway. Inhibition of miR-20b reduced GC cell viability, proliferation and promoted early cell apoptosis; whereas overexpression of miR-451a reduced GC cell growth. These findings reveal the important roles of miR-20b and miR-451a in GC progression, which may be used to develop a beneficial strategy for future cancer therapy.

## 2. Results

### 2.1. Aberrant Expression of miR-20b and miR-451a In Vivo

First, the expression level of miR-20b and miR-451a was determined *in vivo.* For this purpose, biopsy samples of GC patients, healthy controls, and INS-GAS mice samples were analyzed.

**Figure 1:**
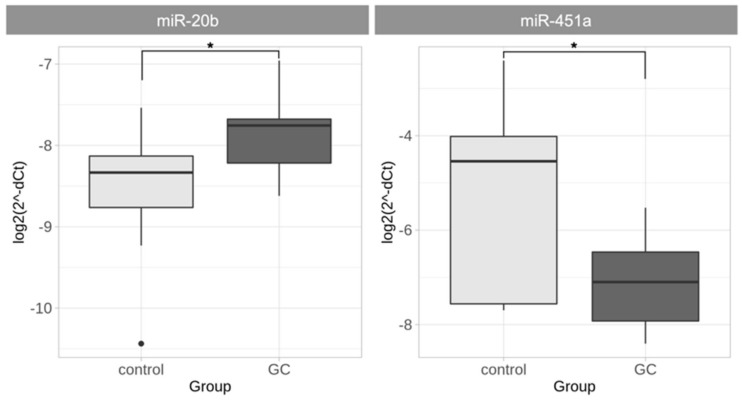
miR-20b and miR-451 expression analysis in GC and control tissues (normalized delta Ct (dCt) values are presented in logarithmic scale). miR-20b expression was significantly increased and miR-451a gene expression was significantly decreased in GC tissue compared to control tissue (* *p* < 0.05).

MiRNA expression analysis in GC samples showed a significant increase of miR-20b (*p* = 0.026) and decrease of miR-451a (*p* = 0.039) expression compared to controls (Figure 1).

**Figure 2:**
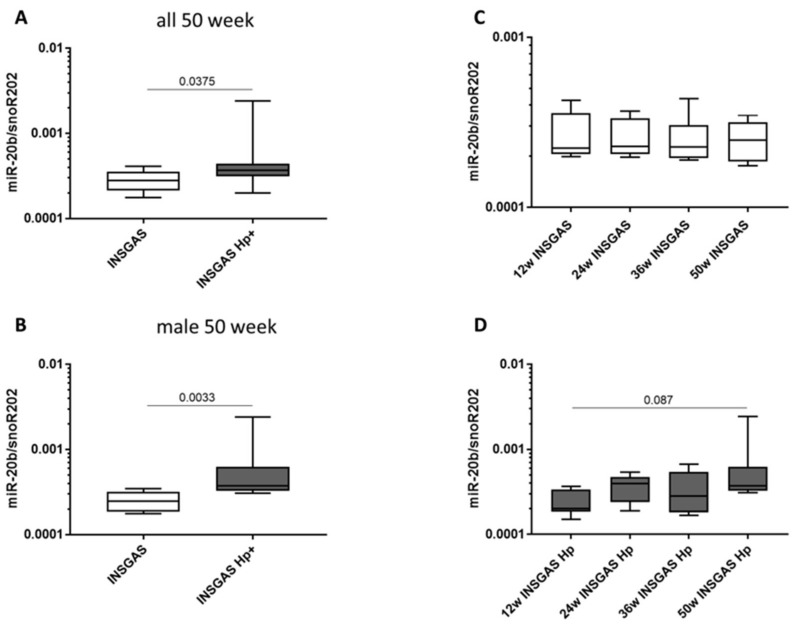
miR-20b expression analysis in vivo using INS-GAS mouse model. (**A**) MiR-20b expression level in male and female mice comparing INS-GAS and *H. pylori* infected INS-GAS mice at 50 weeks. Analysis showed significant increase in miR-20b expression (*p* = 0.0375). (**B**) MiR-20b expression level in male mice comparing INS-GAS and *H. pylori* infected INS-GAS mice at 50 weeks. Results revealed significant increase in miR-20b expression and gender specific expression pattern (*p* = 0.033). (**C** and **D**) MiR-20b expression at 12, 24, 36 and 50 weeks. A stepwise increase in miR-20b expression was determine during the different time points with highest difference at 50 weeks (*p* = 0.003).

Moreover, GC analysis of INS-GAS mice showed a gender specific miR-20b expression pattern following *H. pylori* infection. Only male mice showed significantly higher miR-20b expression for all time points (*p* = 0.029). There was a stepwise increase in miR-20b expression during the different time points from 12 to 50 weeks with the highest difference at 50 weeks (*p* = 0.003) (Figure 2B). No significant changes in miR-451a expression were observed.

### 2.2. Aberrant Expression of miR-20b and miR-451a in Vitro

**Figure 3:**
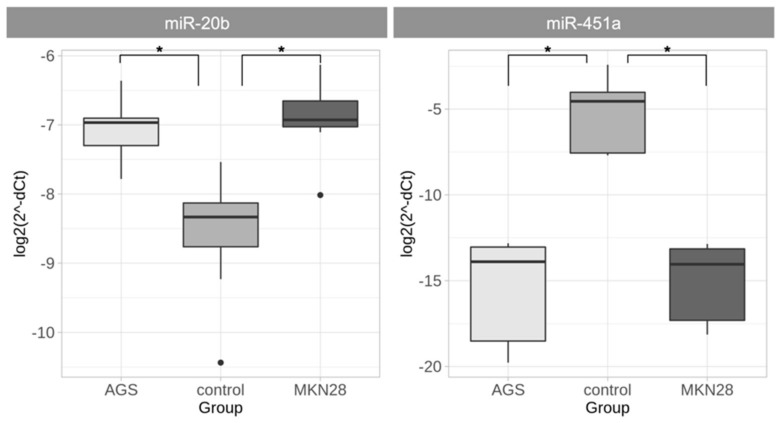
miR-20b and miR-451 expression analysis in AGS and MKN28 cell lines (normalized delta Ct (dCt) values are presented in logarithmic scale). miR-20b expression was significantly increased in both gastric cancer cell cultures compared to control tissue group and miR-451a gene expression was significantly decreased in both gastric cancer cell cultures AGS and MKN28 compared to control tissue group (* *p* < 0.05).

The expression level of miR-20b and miR-451a was also determined in vitro in AGS and MKN28 cell lines compared to normal gastric tissue. Expression level of miR-20b was significantly up-regulated (*p* = 6.35 × 10^−5^ and *p* = 1.03 × 10^−4^, AGS and MKN28, respectively) and miR-451a was down-regulated (*p* = 2.58 × 10^−5^ and *p* = 2.58 × 10^−5^, AGS and MKN28, respectively) in both GC cell lines compared with control gastric tissue (Figure 3). There was no difference on microRNA expression patterns between the two cell lines.

These data suggest that miR-20b may potentially act as onco-miRNA and target tumour-suppressor genes while miR-451a acts as tumour-suppressor miRNA and target proto-oncogenes. Based on the observed expression patterns, mimic of miR-451a and inhibitor of miR-20b were chosen for loss-of-function study for both cell cultures.

### 2.3. Inhibition of miR-20b Reduced Cell Viability and Proliferation

**Figure 4:**
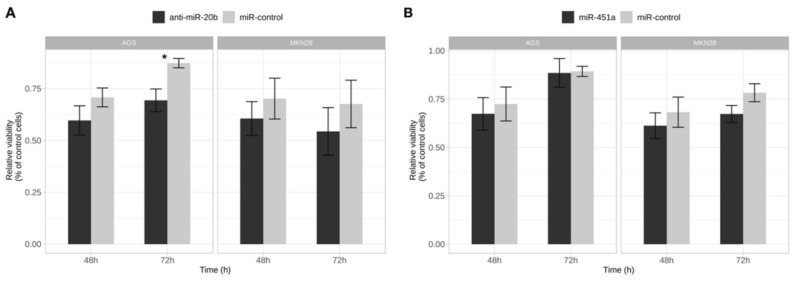
Exogenously altered expression of miR-20b effected cell viability. (**A**) Relative viability changes of AGS and MKN28 cells after inhibition of miR-20b 48 h and 72 h after transfection. Significant results evaluated in AGS cell lines 72h after transfection (* *p* < 0.05); (**B**) Relative viability changes of AGS and MKN28 cells after increased expression of miR-451a 48 h and 72 h after transfection. Data from four independent experiments.

To characterize functional importance of tested miRNAs viability and proliferation of AGS and MKN28 cell lines were tested using MTT assay, 48 h and 72 h after transfection. Reduced cell viability (by 22.1%) was observed in the AGS cell line 72 h after transfection with anti-miR-20b (*p* = 0.029) (Figure 4), whereas no significant changes were observed in MKN28 cell line. Overexpression of miR-451a had no significant effect on viability and proliferation in AGS or MKN28 cells.

### 2.4. Inhibition of miR-20b and Overexpression of miR-451a Dramatically Reduced Colony Formation Rate

**Figure 5:**
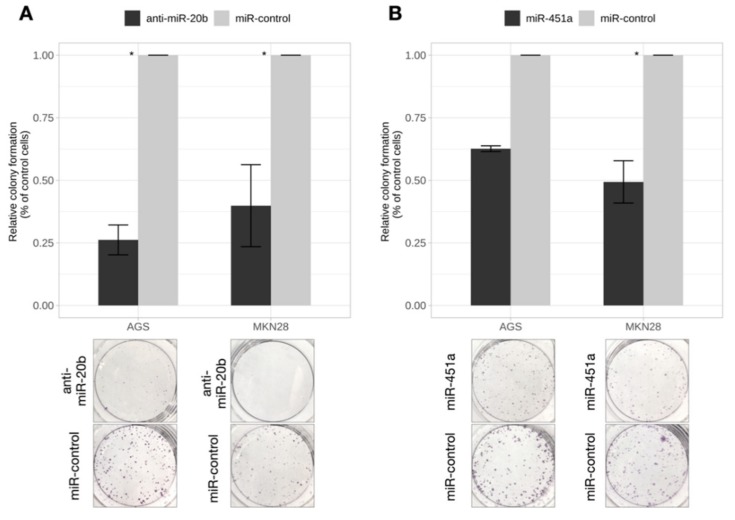
Exogenously altered expression of miR-20b and miR-451a affected colony formation. (**A**) Relative colony formation (of control cells) in AGS and MKN28 cells after inhibition of miR-20b. Formation of colonies reduced significantly in both AGS and MKN28 cell cultures compared to miR-control (* *p* < 0.05); (**B**) Relative colony formation (of control cells) in AGS and MKN28 cells after increased expression of miR-451a. Formation of colonies reduced significantly in both AGS and MKN28 cell cultures compared to miR-control (* *p* < 0.05). Representative pictures of fixed colonies are presented at the bottom of a figure. Data from five independent experiments.

To examine the role of miR-20b and miR-451a in GC cell growth, colony formation assay was conducted. The number of colonies reduced dramatically (AGS by 73.8%; *p* = 2 × 10^−4^ and MKN28 by 60.1%; *p* = 0.021) after inhibition of miR-20b compared to cells transfected with miRNA control (Figure 4). Transfection of GC cells with miR-451a mimic significantly reduced the number of colonies by 50.6% in MKN28 cell culture (*p* = 0.043) (Figure 5).

### 2.5. Inhibition of miR-20b Increased Cell Apoptosis Rate

**Figure 6:**
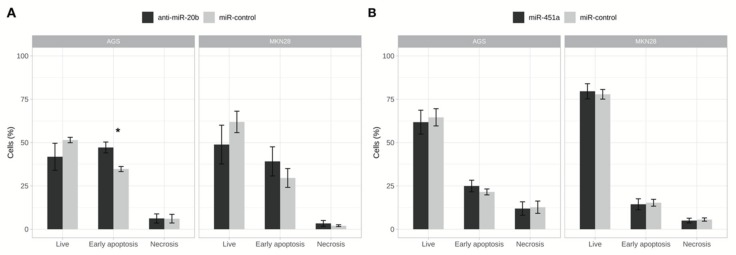
Exogenously altered expression of miR-20b effected proportion of cells undergoing process of early apoptosis. (**A**) Changes of apoptosis rates in AGS and MKN28 cell cultures after inhibition of miR-20b. Proportion of early apoptotic cells increased statistically significantly in AGS cells line (* *p* < 0.05); (**B**) Changes of apoptosis rates in AGS and MKN28 cell cultures after increased expression of miR-451a. Data from three independent experiments.

In order to investigate changes in early apoptosis and cell death rates flow cytometry-based apoptosis assay was employed, where annexin V-FITC positive cells were considered early apoptotic and annexin V-FITC/ PI positive as necrotic cells. A slightly increased rate of apoptotic cells (by 12.5%) was detected in AGS cell culture (*p* = 0.040) after inhibition of miR-20b (Figure 6). However, a decrease in live cells did not reach statistical significance. No effect on live, apoptotic, or necrotic populations was observed in MKN28 cell line after transfection with anti-miR-20b and in both cell cultures after transfection with miR-451a mimic.

### 2.6. miR-20b and miR-451a Have No Effect In Vitro On Cell Migration Processes

The effect of miR-20b and miR-451a on the migration of GC cells was analyzed using wound healing assay. However, no significant changes in migration were determined in both GC cell lines after transfection with anti-miR-20b and miR-451a.

### 2.7. PTEN and TXNIP Are Direct Targets of miR-20b

**Figure 7:**
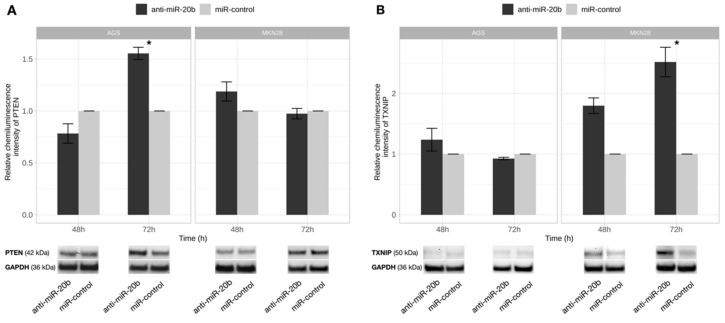
PTEN and TXNIP proteins level changes after exogenous miR-20b expression inhibition. PTEN (**A**) and TXNIP (**B**) protein expression comparison 48 h and 72 h after transfection in AGS and MKN28 cell cultures transfected with anti-miR-20b and miR-control. Significant PTEN protein level increase was determined 72 h after transfection in AGS cell culture and TXNIP- 72 h after transfection in MKN28 cell culture. Representative pictures of PTEN and TXNIP proteins signals detected by Western Blot presented at the bottom of a figure (* *p* < 0.05). Data from four independent experiments.

*IRF1*, *PTEN,* and *TXNIP* were selected in silico as potential target genes of miR-20b. Protein expression analysis revealed that inhibition of miR-20b resulted in a significant increase of PTEN expression 72 h after transfection (*p* = 0.011) in AGS cell line and increase of TXNIP protein expression 72 h after transfection (*p* = 0.025) in MKN28 cells (Figure 7); whereas, no effect on IRF1 protein level was observed in both cell lines.

**Figure 8:**
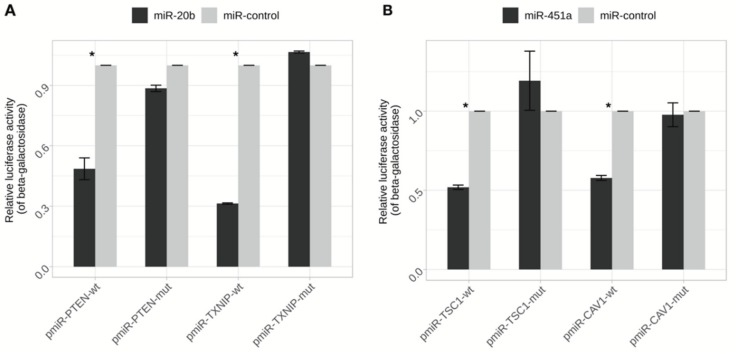
Estimation of direct interaction of investigated miRNAs and predicted target genes 3′ UTR by luciferase reporter assay. (**A**) AGS cell line was cotransfected with miR-20b (or miR-control) and pmiR-PTEN-wt/mut or pmiR-TXNIP-wt/mut vectors. Significant signal decrease was determined in cells transfected with either PTEN or TXNIP wt vectors (* *p* < 0.05); (**B**) AGS cell line was cotransfected with miR-451a (or miR-control) and pmiR-CAV1-wt/mut or pmiR-TSC1-wt/mut vectors. Significant signal decrease was determined in cells transfected with either CAV1 or TSC1 wt vectors (* *p* < 0.05). Luciferase activity was normalized by the beta-galactosidase signals. Results are shown as fold change relative to the negative control. Data from three independent experiments.

To validate the binding specificity of miR-20b to *PTEN* and *TXNIP*, a luciferase reporter system containing 3′ UTR-wt and 3′ UTR-mut regions of the genes was used. AGS cells were co-transfected with reporter vector and miR-20b mimic or negative mimic control. As a result, miR-20b clearly suppressed luciferase activity both in *PTEN*-3′ UTR-wt and *TXNIP*-3′UTR-wt compared to the control (*p* = 0.006 and *p* = 4 × 10^−4^, respectively), whereas firefly luciferase activity did not change for both mut-type vectors (Figure 8). 

### 2.8. miR-451a Directly Regulate the Expression of CAV1 and TSC1

**Figure 9:**
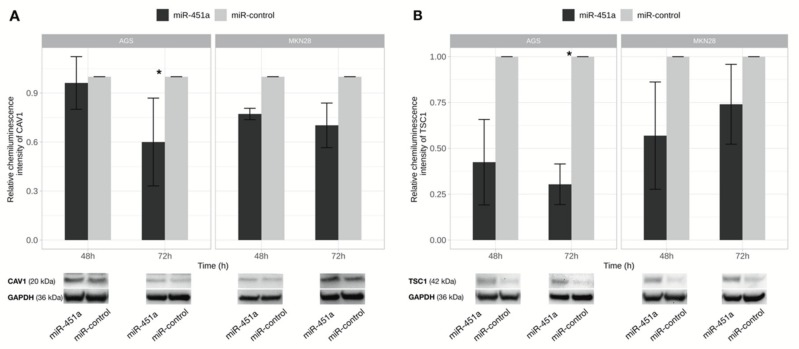
CAV1 and TSC1 proteins level changes after exogenous up-regulation of miR-451a expression. CAV1 (**A**) and TSC1 (**B**) protein expression comparison 48 h and 72 h after transfection in AGS and MKN28 cell cultures transfected with miR-451a and miR-control. Significant CAV1 and TSC1 proteins’ level decrease was determined 72 h after transfection in AGS cell culture. Representative pictures of CAV1 and TSC1 proteins signals detected by Western Blot presented at the bottom of a figure (* *p* < 0.05). Data from three independent experiments.

Using in silico prediction tools as downstream targets of miR-451a, *CAV1* and *TSC1* were selected. Upregulation of miR-451a reduced CAV1 and TSC1 proteins expression in AGS cell culture 72 h after transfection (*p* = 0.011 and *p* = 0.024, respectively) (Figure 9). No significant changes in protein level of target genes was observed in MKN28 cell line. 

Direct binding specificity of miR-451a to *CAV1* and *TSC1* was evaluated using luciferase reporter system containing 3′ UTR-wt and 3′ UTR-mut regions. AGS cells were co-transfected with miR-451a mimic or negative mimic control and reporter vector. The results indicated that miR-451a significantly reduced firefly luciferase activity both in *CAV1*-3′ UTR-wt and *TSC1*-3′UTR-wt (*p* = 0.007 and *p* = 0.009, respectively) compared to the control. Firefly luciferase activity did not change in cells transfected with the mut-type vectors (Figure 8).

## 3. Discussion

Although a number of studies have elucidated miRNA profiles of GC, the role of these miRNAs, their possible target genes and functions remain under investigation. In the present study, we analyzed two miRNAs—miR-20b and miR-451a—shown to be deregulated in many different malignancies, including GC [13,14,15,16]. We determined that miR-20b and miR-451a directly regulate genes involved in the PI3K/AKT/mTOR signaling pathway, and modification of their expression had a tumor-suppressive role in GC.

MiR-20b belongs to a cluster of highly similar miRNAs called the miR-17 family [22]. Deregulation of miR-20b has been determined in many different cancers [13,14,15,23,24,25,26]. Our results demonstrated that miR-20b is overexpressed in both AGS and MKN28 GC cell lines compared to healthy gastric tissue, which is consistent with previous studies [13,14,27,28]. Moreover, our in vitro results were confirmed in vivo in GC samples and INS-GAS mice, showing increased expression of miR-20b in male mice. The INS-GAS mouse model is gender specific and male gastric tissue responds more rapidly and aggressively to *H. pylori* infection due to differences in hormone secretion. Estrogen may protect female mice from intestinal-type tumors. On the other hand, androgens may promote the development of gastric cancer in male mice [29]. Similar pathogenesis patterns have also been observed in humans [30]. 

Using loss-of-function experiments we revealed that miR-20b inhibits proliferation and colony formation abilities, reduces viability, and increases ratio of early apoptotic cells in GC cell lines. These results confirm that miR-20b is highly pronounced as oncogenic, driving cellular processes such as cancer cells proliferation [20] and colony formation [31], invasiveness and tumor growth [19]. Our study is the first which investigates the functional importance of miR-20b in gastric carcinogenesis, GC cell lines, and the GC mouse model INS-GAS.

MiR-451a is located in the 17q11.2, region reported to be amplified in GC and other types of cancers [18]. miR-451a is usually down-regulated and acts as a tumor suppressor in a series of cancers [32,33,34], including GC [16,35]. Our study results have also shown that miR-451a was down-regulated in the investigated GC cell lines. Functional analysis of miR-451a revealed that increased level of this miRNA impaired only colony formation of GC cell lines. A study by Riquelme et al. showed that miR-451a up-regulation in GC could not only affect colony formation but also reduce cell viability [16]. Taking into account other cancer types, increased level of miR-451a has been shown to impair cell growth, moderately reduce migration ability in thyroid carcinoma cells [32], and inhibit migratory and invasive abilities in renal cell carcinoma [34]. MiR-451a may exert its biological role through mediating tumor formation, maintenance, and metastasis.

We have shown that the inhibition of miR-20b reduced viability, colony formation, and increased ratio of apoptotic cells while increased expression of miR-451a only affected colony formation and was inconsistent with other in vitro assays (MTT and Annexin V/Dead Cell Apoptosis). MTT assay reflects that metabolic activity as well as the colony forming assay is considered to show the ability of cells to proliferate, however both of the assays could also be affected by the broader range of the processes in the cells. Annexin V/Dead Cell Apoptosis assay is specifically based on the Annexin V ability to conjugate with phosphatidylserine, which is usually expressed on the cell surface during apoptosis; however, cells could undergo different molecular changes or different types of cell deaths and result in inconsistency between different in vitro assays.

Also, some results of the loss-of-function assays were not consistent between two commercial gastric cancer cell cultures. This may be caused due to a different origin of cell lines: AGS—primary gastric cancer lesion and MKN28—metastatic site in the liver. This could potentially lead to different characteristics of cells in terms of aggressiveness, invasiveness, proliferative potential, or molecular signatures. 

Using in silico analysis, we selected potential target genes of miR-20b: *IRF*, *PTEN,* and *TXNIP* and miR-451a: *CAV1* and *TSC1.* These genes are involved in phosphatidylinositol-3-kinase (PI3K)/AKT/mTOR signaling pathway which has potential prognostic and predictive significance in GC [36,37]. Using Western Blot analysis and luciferase reporter assay we were able to confirm direct *PTEN* and *TXNIP* 3′ UTR interaction with miR-20b and *CAV1* and *TSC1* 3′ UTR interaction with miR-451a.

*PTEN* is a classical tumor suppressor gene in various human cancers. PTEN functions as a negative regulator of the PI3K/AKT pathway through dephosphorylation of phosphatidylinositol 3,4,5 trisphosphate (PIP3), and is involved in regulation of cellular proliferation, metastasis and apoptosis during progression of cancers [38]. PTEN has been reported to be regulated by numerous miRNAs in multiple cancers, including colorectal carcinoma, glioma, ovarian and breast cancer [31,39,40,41]. Previously studies investigated *PTEN* as miR-20b target gene in colorectal and breast cancer [26,31], whereas our study is the first to show *PTEN* as an miR-20b target in GC cells.

*TXNIP* has been identified as potential tumor suppressor gene in various solid tumors and hematological malignancies [42,43,44]. This gene is involved in PI3K/AKT/mTOR pathway by mediation of glucose intake in cancer cells. The changes in glucose metabolism is associated with a great increase of cell bioenergetic and biosynthetic abilities, which are important to maintain rapid cell proliferation, tumor progression, and resistance to chemotherapy and radiation. Oncogenic activation of PI3K/AKT signaling at least partially promotes cellular glucose uptake through the regulation of TXNIP expression [45]. Previous cancer studies have reported that *TXNIP* is regulated by miR-373 [46], miR-411 [47], and miR-224 [48,49]. Our study is the first to show *TXNIP* as an miR-20b target in GC.

CAV1 (caveolin-1) is a structural component of caveolar membrane domains causing the propagation of downstream signals. Stimulation of CAV1-positive cells results in activation of the PI3K/AKT pathway and leads to cell cycle progression through G1 and entry into the S phase [50]. CAV1 is also involved in regulation of PTEN [51]. Previous studies have revealed several miRNAs involved in direct regulation of *CAV1* [52,53,54]. However, our study is the first to indicate miR-451a as a direct regulator of *CAV1*.

TSC1 acts through regulation of the mTOR pathway. Activation of upstream signals results in inhibition of TSC1/TSC2 by AKT, allowing mTOR activation. These changes also result in uncontrolled and increased bioenergetic and biosynthetic processes, cell growth, and proliferation [36]. *TSC1* gene as potential target of miR-451a has already been investigated in GC in a study conducted by Riquelme et al. [16]. However, investigators have not confirmed the direct interaction between miR-451a and *TSC1*. Therefore, our study is the first that has confirmed *TSC1* as target gene of miR-451a.

This study has some potential limitations: although commercial cell lines are derived from human primary or metastatic gastric tumor lesion more appropriate control for analyzing miRNA expression patterns would be culture of normal epithelial cells. However, commercial cell cultures showed similar miR-20b and miR-451a expression pattern compared to patients’ gastric tumor tissue samples. Further studies analyzing the direct impact of miRNA in vivo using xenographic mouse models would be of interest. Moreover, due to the small sample size we were not able to perform association analysis of miR-20b and miR-451a expression and subphenotypes of GC patients (including *H. pylori* infection).

In conclusion, the present study identified that expressions of miR-20b and miR-451a are significantly deregulated in gastric cancer tissue, commercial cell cultures, and INS-GAS mice. Notably, down-regulation of miR-20b and up-regulation of miR-451a expression exhibits an anti-tumor effect in vitro (reduced viability, colony formation, increased apoptosis rate and reduced colony forming ability, miR-20b, and miR-451a respectively) by targeting genes involved in PI3K/AKT/mTOR tumorigenesis signaling pathway. These findings are important for miRNA functional studies in GC and may also help to improve development of new treatment strategies. 

## 4. Materials and Methods

### 4.1. Human Tissue Samples and Cell Lines

Study subject recruitment was conducted at the Department of Gastroenterology, Lithuanian University of Health Sciences (Kaunas, Lithuania) where tumor tissue samples (*n* = 13) were obtained from the primary lesion biopsy. The characteristics of patients are listed in Supplementary Table S1. Gastric tissue samples of a control group (*n* = 13) were collected from healthy subjects without atrophy or intestinal metaplasia based on the Sydney classification. All patients in the control group underwent upper endoscopy with biopsies due to dyspeptic symptoms but had no history of malignancy. The samples were stored at −80 °C as a fresh-frozen sample. The use of biological material in present study was approved by Kaunas Regional Biomedical Research Ethics Committee (protocol no. BE-2-10, 27th May 2011). Each subject has signed written informed consent and all procedures were carried out in accordance with the guidelines of Declaration of Helsinki.

The human gastric adenocarcinoma cell line AGS was obtained from the American Type Culture Collection (ATCC) and MKN28 cell line was kindly provided by Dr. Alexander Link (Department of Gastroenterology, Hepatology and Infectious Diseases, Otto von Guericke University, Germany). Cell cultures were cultivated according to ATCC recommendations. AGS and MKN28 cell lines were cultivated in Ham’s F-12K (Kaighn’s) Medium (GIBCO Invitrogen Life Technologies, Grand Island, NY, USA) and RPMI 1640 medium (GIBCO Invitrogen Life Technologies, Grand Island, NY, USA), respectively. The culture media was supplemented with 10% Fetal Bovine Serum (FBS) (GIBCO Invitrogen Life Technologies, Grand Island, NY, USA) and 1% penicillin-streptomycin solution (5000 U/mL) (GIBCO Invitrogen Life Technologies, Grand Island, NY, USA). Cells were cultured in humidified incubator containing 5% of CO_2_ at 37 °C. Cell lines were tested for mycoplasma contamination using specific primers [55].

### 4.2. Cell Transfection

MiRNA mimics of miR-451a and miR-20b, miRNA inhibitor of miR-20b (anti-miR-20b), and non-specific miRNA mimic or inhibitor negative control (miRVana^TM^, Ambion by Thermo Fisher Scientific, Grand Island, NY, USA) were used for the transfection of AGS and MKN28 cell lines. Transfection was performed using Lipofectamine 3000 transfection reagent (Thermo Fisher Scientific, Waltham, MA, USA) in accordance with the manufacturer’s recommendations. A final concentration of 90 nM of anti-miR-20b for AGS cell line, 120 nM of anti-miR-20b for MKN28 cell line and a final concentration of 50 nM of miR-451a mimic for both cell lines was used. Efficiency of transfection was tested and monitored by using positive transfection controls let-7c and miR-1 (miRVana^TM^, Ambion by Thermo Fisher Scientific, Grand Island, NY, USA), and fluorescent siRNA (BLOCK-iT^TM^ Alexa Flour Red Fluorescent, Thermo Fisher Scientific, Waltham, MA, USA).

### 4.3. Target Prediction

GC-associated putative target-genes of selected miRNAs were retrieved from databases *(DIANA Lab Tools TarBase*, *miRanda*, *TargetScan*) according to their function in carcinogenesis (oncogenes or tumour-suppressor genes). Three potential target genes (*IRF1, TXNIP, PTEN*) with a proto-oncogenic function were selected for miR-20b and two (*CAV1*, *TSC1*) with an onco-suppressor function for miR-451a.

### 4.4. qRT-PCR for miR-20b and miR-451a Expression Level

To estimate miRNA expression level total RNA from GC (*n* = 13), normal gastric tissue (*n* = 13), and GC-derived cell lines (*n* = 7) was isolated using miRNeasy Micro Kit (Qiagen, Hilden, Germany) according to manufacturer’s protocol. Expression level of miR-20b and miR-451a was determined using TaqMan miRNA assays (Ambion by Thermo Fisher Scientific, Grand Island, NY, USA) using miRNA-specific primers on 7500^TM^ Fast real-time PCR system (Life Technologies, Carlsbad, CA, USA). Levels of miRNA were normalized to miR-16 and changes in expression were calculated using 2^−∆Ct^ method. 

### 4.5. Western Blot

AGS and MKN28 cells (250,000 and 200,000 cells/well, respectively) were transfected with mimic of miR-451a, inhibitor of miR-20b and respective miRNA controls for 48 h and 72 h. Total protein from cells was lysed in 1× radioimmunoprecipitation assay (RIPA) buffer (Abcam, Cambridge, UK) containing protease and phosphatase inhibitor cocktail (Sigma Aldrich, St. Louis, MO, USA). Protein concentration was estimated using Pierce BCA Protein Assay Kit (Thermo Scientific, USA). Total protein was separated by SDS-PAGE using 4–12% Bis-Tris Plus Mini Gels and blotted to 0.45 µm PVDF membrane. Membranes were blocked in WesternBreeze Blocker/Diluent (Part A and B) (Thermo Fisher Scientific, Waltham, MA, USA) at room temperature for 1 h. Antibodies directed against IRF1 (1:1000 dilution; Cat. No. ab186384; Abcam, Cambridge, UK), PTEN (1:2500 dilution; Cat. No. ab32199, Abcam, Cambridge, UK), TXNIP (8 µg/mL concentration; Cat. No. 40-3700; Thermo Fisher Scientific, Waltham, MA, USA), CAV1 (1:500 dilution, Cat. No. ab192869, Abcam, Cambridge, UK), TSC1 (1:2000 dilution, Cat. No. 37-0400, Thermo Fisher Scientific, Waltham, MA, USA), and GAPDH (0.4 µg/mL concentration; Cat. No. AM4300; Ambion by Thermo Fisher Scientific, Grand Island, NY, USA) were used. The signals were visualized with ChemiDoc XRS+ System (Bio-Rad, Hercules, CA, USA) and ImageLab Software (version 5.2.1, (Bio-Rad, Hercules, CA, USA). Protein levels were normalized to endogenous control of GAPDH protein.

### 4.6. Luciferase Reporter Assay

Wild-type (wt) and mutant (mut) seed regions of the target genes (*PTEN*, *TXNIP*, *TSC1* and *CAV1*) were constructed and cloned into Luc 3′UTR between the HindIII and BcuI sites of pMIR-REPORT-Luciferase vector (Ambion by Thermo Fisher Scientific, Grand Island, NY, USA). The oligonucleotide sequences are listed in Supplementary Table S2. Constructed vectors were verified by Sanger sequencing using Applied Biosystems^®^ 3500 analyzer (Applied Biosystems, Foster City, CA, USA). AGS cells (100,000 cells/well) were co-transfected with 146 ng of constructs (wt or mut vector) and with 50 nM of either miRNA mimic or negative mimic control using Lipofectamine 3000 (Thermo Fisher Scientific, Waltham, MA, USA). After 48 h incubation luciferase activity was detected by Dual-Light™ Luciferase & β-Galactosidase Reporter Gene Assay System (Applied Biosystems, Foster City, CA, USA) following manufacturer’s protocol. Luminescent signal was quantified by GENios Pro microplate reader (Tecan Trading AG, Mannedorf, Switzerland). Reporter activity was normalized to β-Galactosidase activity.

### 4.7. MTT Assay

Viability and proliferation of AGS and MKN28 cells (4000 and 3500 cells/well, respectively) was measured with 3-(4,5-dimethylthiazol-2-yl)-2,5-diphenyltetrazolium bromide (MTT) assay 48 h and 72 h after transfection. MTT reagent (final concentration 5 mg/mL) (Sigma Aldrich, St. Louis, MO, USA) was added to cells and incubated for 2 h at 37 °C. After incubation supernatant was discarded and formazan was dissolved in 200 µl dimethyl sulfoxide (DMSO) (Sigma Aldrich, St. Louis, MO, USA). Optical density (OD) was determined by Sunrise absorbance microplate reader (Tecan Trading AG, Mannedorf, Switzerland) at wavelength 570 nm and 620 nm (as reference).

### 4.8. Colony Formation Assay 

Both GC cancer cell lines (750 and 1000 cells/well, AGS and MKN28, respectively) were seeded in duplicates onto 6-well plates and transfected after 24 h. After 9 days of incubation of AGS cells and 8 days of incubation of MKN28, cells were washed with PBS, fixed with 10% formalin, and stained with 0.5% crystal violet. Colonies were counted using ImageJ software (version 1.52g).

### 4.9. Apoptosis Assay

Apoptosis of cells (100,000 cells/well) was measured using FITC Annexin V/Dead Cell Apoptosis Kit with FITC Annexin V and PI (Invitrogen, Carlsbad, CA, USA) 72 h after transfection. Harvested cells were centrifuged and suspended in 1× Annexin binding buffer. Cell suspension was incubated with Annexin V-FITC and PI according to manufacturer’s protocol. Samples were analyzed using Accuri C6 flow cytometer (BD Biosciences, Erembodegem, Belgium). Cells were discriminated into viable (both annexin V-FITC/ PI negative), apoptotic (annexin V-FITC positive), and dead cells (both annexin V-FITC/ PI positive).

### 4.10. Wound Healing Assay

Migration of transfected AGS and MKN28 (100,000 and 75,000 cell/well, respectively) was determined using wound healing assay. Cells were transferred to two well culture-inserts (Ibidi, Munich, Germany) 24 h after transfection. After the overnight incubation, cell culture inserts were removed to create 500 µm gap between the cells. Cell migration was monitored at 0 h, 24 h, and 48 h after the removal of culture-inserts under IX71 microscope (Olympus, Tokyo, Japan).

### 4.11. Tumorigenicity of miR-20b and miR-451a in INS-GAS Mouse Model

All in vivo experiments were carried out on the insulin-gastrin (INS-GAS) transgenic mouse model. Model was used to evaluate the alterations of miR-20b and miR-451a expression following *H. pylori* infection with a follow-up from 12 to 50 weeks of age.

### 4.12. Statistical Analysis

Experimental data is presented as means ± standard deviation (SD) of three to five independent experiments. All analyses were performed with R Studio software (R version 3.3.3); a value of *p* < 0.05 was considered statistically significant. Data distribution was determined by the Shapiro–Wilk test, which was used to determine whether the data distribution was normal or non-normal. The *T*-test was used to determine significance of difference between data with normal distribution. The Wilcoxon test was used for significance of difference between data with non-normal distribution. 

## Supplementary Materials

The following are available online at https://www.mdpi.com/1422-0067/21/3/877/s1, Table S1: Characteristics of gastric cancer patients and controls. Table S2: binding positions and sequences of inserts for investigation of miRNA direct binding to target genes (*PTEN, TXNIP, CAV1* and *TSC1*) by luciferase reporter assay.

## Abbreviations

3′ UTR	three prime untranslated region
ATCC	American type culture collection
CAV1	caveolin-1
DMSO	dimethyl sulfoxide
FBS	fetal bovine serum
FITC	fluorescein isothiocyanate
GAPDH	glyceraldehyde 3-phosphate dehydrogenase
GC	gastric cancer
HER	human epidermal growth factor receptor
INS-GAS	insulin-gastrin
IRF1	interferon regulatory factor 1
miRNA	microRNA
mTOR	mammalian target of rapamycin
MTT	3-(4,5-dimethylthiazol-2-yl)-2,5-diphenyltetrazolium bromide
mut	mutant
OD	optical density
PI	propidium iodide
PI3K	phosphatidylinositol-3-kinase
PTEN	phosphatase and tensin homologue
RIPA	radioimmunoprecipitation assay
RISC	RNA-induced silencing complex
RNA	ribonucleic acid
TSC1	tuberous sclerosis 1
TXNIP	thioredoxin-interacting protein
wt	wild-type
