# Supplementary material for: miR-20b and miR-451a Are Involved in Gastric Carcinogenesis through the PI3K/AKT/mTOR Signaling Pathway: Data from Gastric Cancer Patients, Cell Lines and Ins-Gas Mouse Model
Source: Int J Mol Sci. 2020 Jan 29;21(3):877. doi: 10.3390/ijms21030877 (PMC7038213; doi:10.3390/ijms21030877)
Supplement: Supplementary file 1 [file ijms-21-00877-s001.pdf]

Table S1: Characteristics of gastric cancer patients and controls.

	**Gastric cancer patients** (n = 13)	**Controls** (n = 13)
**Age,** mean ± SD	68 ± 12.7	63 ± 11.9
**Gender,** n (%)		
Male	8 (61.5)	5 (38.5)
Female	5 (38.5)	8 (61.5)
H. Pylori* status, n (%)		
Positive	8 (61.5)	2 (15.4)
Negative	3 (23.1)	10 (76.9)
Unknown	2 (15.4)	1 (7.7)
**Differentiation grade**, n (%)		
G1	2 (15.4)	-
G2	3 (23.1)	-
G3	8 (61.5)	-
**Lauren**, n (%)		
Diffuse	7 (53.8)	-
Intestinal	6 (46.2)	-

* Anti-*Helicobacter pylori* IgG ELISA test

Table S2: binding positions and sequences of inserts for investigation of miRNA direct binding to target genes (*PTEN*, *TXNIP*, *CAV1* and *TSC1*) by luciferase reporter assay.

**miRNA binding position**	272-278		796-803		1883-1890		154-160	
**Insert 3'-- --5' (Reverse) sequence**	TTTATTAAGCT<br>TTAAAACTGG<br>AATAAAACGG<br>GAAAGTGCCA<br>TCTTTATTAAT<br>CCTAATTACTA<br>GTGCGCAG	TTTATTAAG<br>CTTTAAAAC<br>TGGAATAAA<br>ACGGGAAA<br>ACGCCATCT<br>TTATTAATC<br>CTAATTACT<br>AGTGCGCAG	TTTATTAAG<br>CTTACAAAA<br>AATTTGACA<br>GGAATTAAA<br>GTGCTAAGA<br>ACATCACCT<br>TAGAATACT<br>AGTGCGCAG	TTTATTAAG<br>CTTACAAAA<br>AATTTGACA<br>GGAATTAAA<br>GCACTAAGA<br>ACATCACCT<br>TAGAATACT<br>AGTGCGCAG	TTTATTAAGCT<br>TCAACAACAA<br>GGCCGACTGT<br>TTAACCGTTAA<br>ACATTTTTATT<br>GTGCAGGACT<br>AGTGCGCAG	TTTATTAA<br>GCTTCAAC<br>AACAAGG<br>CCGACTGT<br>TTAATAGT<br>TAAACATT<br>TTTATTGT<br>GCAGGACT<br>AGTGCGCA<br>G	TTATTAAGC<br>TTATTTCAA<br>TGCCAGATC<br>CAAAAACCG<br>TTCTGCATT<br>CAGTCAGCT<br>GTCCACTAG<br>TGCGCA	TTTATTAAGCT<br>TATTTCAATGC<br>CAGATCCAAA<br>AACTATTCTGC<br>ATTCAGTCAG<br>CTGTCCACTA<br>GTGCGCAG
**Insert 5'-- --3' (Forward) sequence**	CTGCGCACTA<br>GTAATTAGGA<br>TTAATAAAGA<br>TGGCACTTTCC<br>CGTTTTATTCC<br>AGTTTTAAAG<br>CTTAATAAA	CTGCGCACT<br>AGTAATTAG<br>GATTAATAA<br>AGATGGCGT<br>TTTCCCGTTT<br>TATTCCAGT<br>TTTAAAGCT<br>TAATAAA	CTGCGCACT<br>AGTATTCTA<br>AGGTGATGT<br>TCTTAGCAC<br>TTTAATTCCT<br>GTCAAATTT<br>TTTGTAAGC<br>TTAATAAA	CTGCGCACT<br>AGTATTCTA<br>AGGTGATGT<br>TCTTAGTGC<br>TTTAATTCCT<br>GTCAAATTT<br>TTTGTAAGC<br>TTAATAAA	CTGCGCACTA<br>GTCCTGCACA<br>ATAAAAATGT<br>TTAACGGTTA<br>AACAGTCGGC<br>CTTGTTGTTGA<br>AGCTTAATAA<br>A	CTGCGCAC<br>TAGTCCTG<br>CACAATAA<br>AAATGTTT<br>AACTATTA<br>AACAGTCG<br>GCCTTGTT<br>GTTGAAGC<br>TTAATAAA	TGCGCACTA<br>GTGGACAGC<br>TGACTGAAT<br>GCAGAACGG<br>TTTTTGGAT<br>CTGGCATTG<br>AAATAAGCT<br>TAATAA	CTGCGCACTA<br>GTGGACAGCT<br>GACTGAATGC<br>AGAATAGTTTT<br>TGGATCTGGC<br>ATTGAAATAA<br>GCTTAATAAA
**Insert type**	wt	mut	wt	mut	wt	mut	wt	mut
**Target Ensembl number**	ENSG00000171862		ENSG00000265972		ENSG00000105974		ENSG00000165699	
**Target symbol**	*PTEN*		*TXNIP*		*CAV1*		*TSC1*	
**Mature miRNA ID**	hsa-miR-20b		hsa-miR-20b		hsa-miR-451a		hsa-miR-451a	
**Mature miRNA accession number**	MIMAT0001413		MIMAT0001413		MIMAT0001631		MIMAT0001631	
